# Managing Chronic Regional Pain Syndrome: The Potential Impact of the Food and Drug Omnibus Reform Act

**DOI:** 10.7759/cureus.38336

**Published:** 2023-04-30

**Authors:** Andrew B Herson, Chase W Thompson, Cody A Barbari, Steven T Fischer, Gina M Rehm, Brooke T Miller, David M Herson

**Affiliations:** 1 Pain Management, Lake Erie College of Osteopathic Medicine, Bradenton, USA; 2 Physical Medicine and Rehabilitation, Memorial Hospital, Hollywood, USA; 3 Pain Management, David Herson MD PA, Tampa, USA

**Keywords:** chronic pain, budapest criteria, upper extremity injury, type 1 crps, food and drug omnibus reform act, chronic regional pain syndrome, off-label treatments

## Abstract

Complex regional pain syndrome (CRPS) is a neurological disorder characterized by persistent limb symptoms. With there being no definitive tests, diagnosis can be challenging. The Budapest criteria are the standard for diagnosis. The underlying mechanisms of CRPS involve changes in skin innervation, sensitization of the nervous system, inflammatory cytokines, and genetic and psychological factors. Treatment typically involves a multidisciplinary approach. We present a case of a 71-year-old male with CRPS involving the right upper extremity and a complex history of management including physical therapy, oxycodone, muscle relaxers, non-steroidal anti-inflammatory drugs, and multiple stellate ganglion blocks. The patient manages his pain with off-label medications, including methadone, duloxetine, and pregabalin. In the United States, the management of chronic pain may be affected by potential usage restrictions imposed by the Food and Drug Omnibus Reform Act (FDORA). Under this new act, physicians may face limitations in prescribing off-label medications for specific diagnoses. We aim to highlight the need for prioritizing patient care and individualized treatment in healthcare policy decision-making.

## Introduction

Complex regional pain syndrome (CRPS) is a persistent neurological condition that affects the limbs [1]. It is described as severe pain, swelling, blood vessel regulation and sweating abnormalities, and reduced motor function [1]. CRPS can be classified into two types: type 1 (without nerve injury) and type 2 (with nerve injury) [2]. CRPS is a rare disorder, with a prevalence of <2% in most studies [1]. Clinical diagnosis of CRPS is based on the presence of symptoms such as severe pain, swelling, and changes in skin temperature/color. Imaging studies may also be useful in ruling out other conditions and confirming the diagnosis of CRPS [1]. The pathophysiology of CRPS is hypothesized to involve abnormal activation of the sympathetic nervous system, inflammation, and changes in blood flow [3,4]. Several studies have suggested that CRPS may be related to immune dysfunction, with evidence of increased levels of pro-inflammatory cytokines and other immune markers in patients with the condition [3,4].

The management of CRPS can be quite difficult. Initially, conservative measures are best. Management includes medications, physical therapy, and procedures to alleviate pain and enhance function [5]. The use of pharmacological methods like bisphosphonates, glucocorticoids, and vasoactive mediators appears to be the most successful approach, particularly when paired with physical and occupational rehabilitation [6]. In certain situations, more intrusive interventions like nerve blocks, spinal cord stimulation, or sympathectomy may be utilized. There is an ongoing debate regarding the efficacy and safety of these treatments and further research is warranted to determine their long-term effectiveness and safety [7]. Currently, there are no Food and Drug Administration (FDA)-approved medications for the treatment of CRPS [8]. As such, treatment regimens often necessitate the use of off-label medications, which if limited, could leave providers and patients with fewer options to manage their symptoms.

The Food and Drug Omnibus Reform Act (FDORA) is legislation that provides the FDA the power to restrict the off-label use of medications and therapies. Currently, the scope of the FDORA is an encroachment on the patient-physician relationship and could lead to fewer options for patients in the management of chronic conditions like CRPS. It is vital that this bill be enacted with caution and its scope more clearly defined to not inhibit patient care.

## Case presentation

A 71-year-old male with a past medical history of osteoarthritis, obesity, multiple surgeries on his right upper extremity, and CRPS presented to our clinic with right wrist and forearm pain. In 1999, the patient fell off a ladder and fractured his right wrist. He suffered ulnar nerve damage and developed CRPS in the right wrist/forearm with chronic intractable pain.

He described the pain as burning, stinging, and throbbing. He rated the pain 6/10 on the visual analog scale (VAS), which was constant. He stated the pain had increased to an 8/10 in severity over the past month. He admitted to weakness, numbness, and tingling in his right arm/wrist. Initial vital signs on presentation were within normal limits. Laboratory tests were not indicated. Recent imaging, including cervical X-ray, showed degenerative changes at C5-C6 primarily consistent with anterior spurring. The venous Doppler of his right arm was negative for deep venous thrombosis. Prior treatments include extensive physical therapy, non-steroidal anti-inflammatory drugs (NSAIDs), muscle relaxers, oxycodone, and multiple stellate ganglion block, all of which failed to give him adequate pain relief.

On physical exam, the patient had erythematous discoloration, swelling, and a shiny appearance of the skin of the right distal forearm and wrist. There was also increased hair growth on the right forearm and wrist as compared to the left. Right-hand grip strength was 4/5. The patient had sufficient Budapest criteria to diagnose CRPS. If there was any doubt, then either a triple-phase bone scan or a thermogram could have been employed. The patient takes methadone 10 mg in the morning, 5 mg at noon, and 5 mg at night, duloxetine 60 mg daily, and pregabalin 100 mg twice a day. The duloxetine in this patient is being used solely for the treatment of his neuropathic pain. This patient does not have depression or any other psychiatric conditions. Breakthrough pain was brought on by using his right upper extremity, gripping, twisting, or rub-on-rub-off motion. The patient remained compliant with his pain medication regimen and monthly routine follow-up appointments.

## Discussion

CRPS is a neurological disorder that results in persistent symptoms in the limbs [1]. These symptoms can include intense pain, inflammation, blood vessel regulation and sweating irregularities, and decreased motor function [2]. CRPS has an estimated occurrence rate of around 5.4 x 10^26.2 per 100,000 person-years [9]. Based on epidemiological patterns, there is evidence to indicate that being female, experiencing an upper extremity injury, or sustaining high-energy trauma can elevate the risk of developing this condition [9]. There are two types of CRPS: type 1, which occurs without nerve injury, and type 2, which occurs with nerve injury [1]. As our patient experienced trauma to his right arm, he was diagnosed with CRPS type 2.

**Figure 1:**
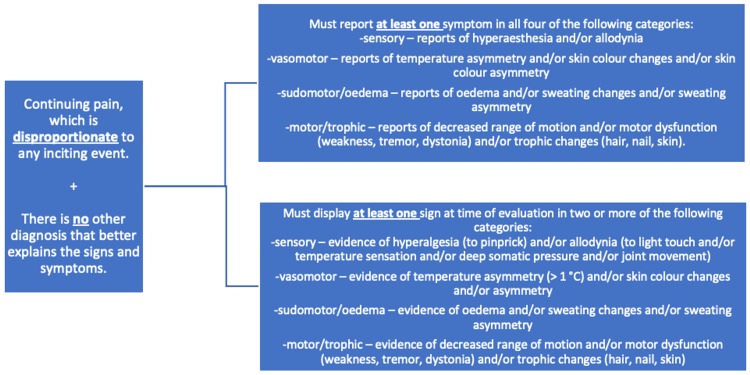
Budapest criteria for complex regional pain syndrome (CRPS) diagnosis Adapted from [9].

CRPS commonly presents with allodynia, hyperalgesia, skin temperature changes, and edema [9]. Currently, there are no definitive confirmatory tests for CRPS, making diagnosis challenging [9]. In 2003, a revised set of diagnostic criteria, commonly referred to as the Budapest criteria (Figure 1), was established and is considered the standard for diagnosing CRPS [9,10].

**Figure 2:**
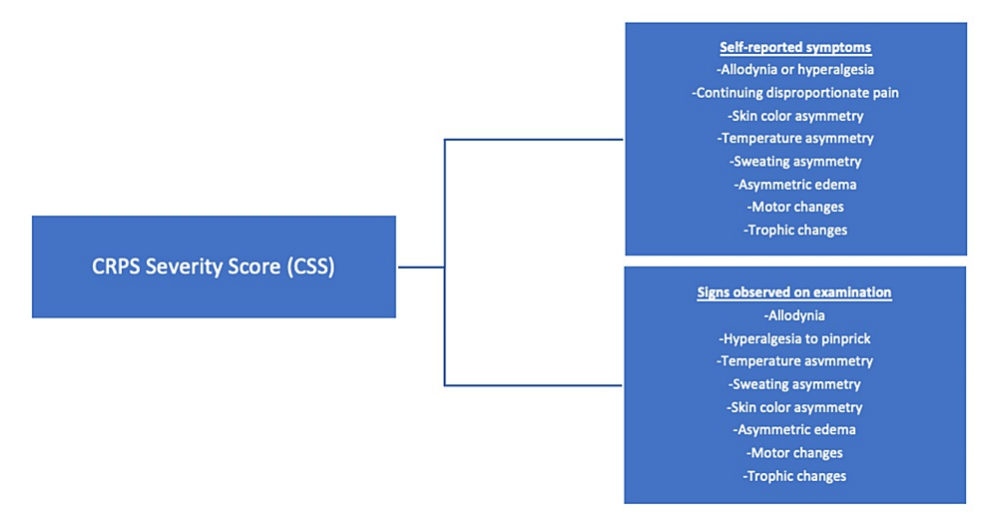
CRPS severity score (CSS) The maximum CSS score is 16. Each symptom and sign is assigned a score of 1 for assessment. Higher scores indicate greater CRPS severity. Lower scores indicate lesser CRPS severity [11]. CRPS: complex regional pain syndrome.

The CRPS severity score (CSS) is a tool devised by Harden et al. (2010) to evaluate the severity of CRPS (Figure 2) [11]. It encompasses 16 items that reflect various CRPS symptoms, including pain, sensory alterations, motor dysfunction, swelling, and temperature changes [11]. The CSS has been used as an objective clinical tool to measure the severity of CRPS and track changes in symptoms over time. This includes the response to treatment interventions [11].

The underlying mechanisms that are thought to contribute to CRPS involve changes in skin innervation, such as a decrease in the density of small nerve fibers (C and Aα) [1]. There is also central and peripheral sensitization, which involves increased excitability of pain-sensing neurons in the spinal cord and local tissues due to persistent pain signals from tissue damage or nerve injury [1,12]. This sensitization is mediated by neuropeptides like substance P and bradykinin [1]. Additionally, there is altered function of the sympathetic nervous system, lower levels of circulating catecholamines, increased levels of inflammatory cytokines (tumor necrosis factor-α, interleukin 1, 2, and 6) both locally and systemically, lower systemic levels of anti-inflammatory cytokines (interleukin-10), genetic factors (human leukocyte antigen (HLA)-B62 and HLA-DQ8 alleles), and psychological factors such as anxiety, anger, and depression [1,3,13].

Given the wide symptom range and complexity of CRPS, patients often require input from multiple specialties [14]. The approach to the treatment of CRPS usually involves a combination of physical therapy, medications, and other interventions with the goal to reduce pain and improve function [5]. This approach is different for each patient and consists of trial and error of various medications, the effectiveness of which is subjectively reported by the patient. The lack of a concise approach highlights the difficulty and complexity of the management of CRPS. Bisphosphonates, glucocorticoids, and vasoactive mediators appear to be the most effective pharmacological interventions. Medication is often accompanied by physical and occupational rehabilitation [6]. Treatment differs between patients, as everyone may have a different response to interventions. While not approved for CRPS, some medications may still be used off-label to manage the symptoms and alleviate associated pain. Off-label medications typically used in CRPS include serotonin-norepinephrine reuptake inhibitors (SNRIs), gabapentin or pregabalin, and lidocaine ointment.

With there being no specifically approved medications for this condition, providers are challenged with a selection of medications for the treatment of CRPS with there being limited high-quality evidence to support their use [8].

SNRIs such as duloxetine and venlafaxine can be used to relieve pain, improve sleep, and manage mood symptoms in patients with CRPS [9]. Gabapentin and pregabalin commonly treat neuropathic pain involved with CRPS [9]. Typically used adjunctively, topical lidocaine has been used in the treatment of CRPS [15]. More invasive treatments such as nerve blocks, spinal cord stimulation, and sympathectomy are used in some cases of CRPS [16]. Reducing the over-activity of the sympathetic nerves seen in CRPS, stellate ganglion blocks are primarily indicated for treatment [17]. However, the effectiveness of these treatments remains controversial. Additional research is required to determine their long-term safety and efficacy [16].

Food and Drug Omnibus Reform Act (FDORA)

In the United States, the use of off-label medications refers to the practice of prescribing medications for conditions or uses other than those indicated by the FDA [18]. Although off-label use of medications is legal and common, it has been viewed as controversial and can carry some risks. [18]. Physicians often prescribe off-label medications when they believe they could benefit the patient, especially when first-line interventions fail [8,18]. New legislation in the FDORA has amended the Food, Drug, and Cosmetic Act (FDC Act), giving the FDA the power to ban a physician’s ability to prescribe off-label medications and medical devices [19]. Meaning, a physician might think a medication could be beneficial for their patient but will not be able to prescribe it.

The recent changes introduced in FDORA could have significant implications for patients relying on off-label treatments. Our patient relies on medications like duloxetine, methadone, pregabalin, and 5% lidocaine ointment - medications that could all be directly affected. This could cause patients to face limited access to care and be denied treatment. Potential interference in the physician-patient relationship could negatively impact the care a patient receives [19]. Depriving patients of potentially beneficial treatments due to FDA restrictions on off-label use could result in unwarranted intrusions into the practice of medicine, especially for conditions like CRPS where off-label medication use is common and FDA-approved options are nonexistent.

The enactment of FDORA should be done so with caution and its use limited to specific, necessary cases. The scope of the bill must be more clearly defined, whether through an establishment of specific criteria that must be met before an off-label intervention becomes restricted, or better definitions of the regulatory power and restriction of this bill. There should also be consideration of an official listing of interventions with their respective off-label uses, whether affiliated with the FDA or another regulatory agency. This listing could allow better documentation of off-label uses of medications and interventions that may lack necessary evidence for FDA indication but have enough observed efficacy in the clinical setting to warrant off-label use, though this is not without its own challenges.

## Conclusions

Our case presents an interesting perspective on the United States FDORA and its potential implications on physicians' ability to care for their patients. We aim to highlight the potential consequences of this legislation on individuals suffering from CRPS who depend on off-label medications for the management of their chronic condition. Our patient, like many others, has found relief and improved quality of life through off-label interventions. Limiting access to these therapies could significantly restrict treatment options, possibly completely, and negatively impact patients' lives. Therefore, we believe that it is imperative that lawmakers evaluate the impact of the FDORA on the physician-patient relationship and take necessary measures to safeguard patients' access to off-label treatments. Prioritizing patient care and outcomes along with individualized treatment should be a top priority when healthcare policy legislation changes are made.
